# Retraction: Shin, J.-H.; Jeong, C.-W. Zipper Is Necessary for Branching Morphogenesis of the Terminal Cells in the *Drosophila melanogaster*’s Tracheal System. *Biology* 2021, *10*, 729

**DOI:** 10.3390/biology11071038

**Published:** 2022-07-11

**Authors:** Jong-Hyeon Shin, Chan-Woo Jeong

**Affiliations:** 1 Department of Molecular Biology, Princeton University, Princeton, NJ 08544, USA; 2 Department of Medicine, Seoul National University, Gwanak-gu, Seoul 08826, Korea; happysmily@jfclinic.com

The published article [1] has been retracted at the request of the authors. Following publication, the authors contacted the editorial office regarding errors in the data and methodology.

Although Figure 3 describes a validation experiment performed using an alternative stock of *D. melanogaster*—more specifically virgin female fruit flies with *btl > gal4*, *UAS > cytoplasmic gfp* and male stock with *btl > gal4*, *UAS > zipper RNAi* genes—following publication the authors realized that these were not independent stock from the initial one, but rather fruit flies stored in another container. Therefore, this was not a validation experiment and the entire dataset in Figure 3 is illegitimate. Furthermore, although Figure 4 is a rescue experiment demonstrating the sufficiency of Zipper in branching morphogenesis, the term ‘rescue experiment’ is inaccurate. A rescue experiment means that a gene-loss individual is able to reobtain a particular phenotype with the addition of such gene/protein. Yet, the methodology simply used an alternative stock that had the protein Zipper. Thus, there is no clear evidence of RNAi-induced fruit flies reobtaining the branching phenotype.

This retraction was approved by the Editor-in-Chief of the journal. The authors agreed to this retraction.
